# Editorial: EMG/EEG Signals-Based Control of Assistive and Rehabilitation Robots

**DOI:** 10.3389/fnbot.2022.840321

**Published:** 2022-02-07

**Authors:** R. A. R. C. Gopura, Thilina Dulantha Lalitharatne, Kazuo Kiguchi, Dingguo Zhang

**Affiliations:** ^1^Department of Mechanical Engineering, University of Moratuwa, Moratuwa, Sri Lanka; ^2^Department of Medical Technology, University of Moratuwa, Moratuwa, Sri Lanka; ^3^Dyson School of Design Engineering, Imperial College London, London, United Kingdom; ^4^Department of Mechanical Engineering, Kyushu University, Fukuoka, Japan; ^5^Department of Electronic and Electrical Engineering, University of Bath, Bath, United Kingdom

**Keywords:** EMG signal, EEG signal, assistive robot, rehabilitation robot, robot control (RC)

Assistive robots support persons with disabilities to improve their quality of life and independence. These robots make the life of a physically weak person more comfortable and useful to society. They sense, process sensory information, and execute actions that are helpful to the disabled person. Similarly, these robots carry out rehabilitation by understanding and augmenting the rehabilitation process through a robotic system. These robots are expected to assist different sensorimotor functions, therapeutic training, and assessment of the sensorimotor performance of the patients.

Assistive and rehabilitation robotic technologies have developed rapidly expanding the horizon of the field of robotics. These technologies are vital in the society in which around one billion among the growing global population experience some form of physical disability impacting everyday life. Assistive and rehabilitation robotic research, especially exoskeleton robotic research integrates advanced mechatronics and intelligent sensing to restore weak sensorimotor functions. Controlling these robots according to the requirement is one of the challenging tasks. Electromyography (EMG) signals have extensively been used to control assistive robots such as exoskeleton robots so that the wearer is free of actuating any additional device to control the robot. Additionally, many studies have been carried out recently to investigate the possibility of using Electroencephalography (EEG) signals to control robotic devices.

This Research Topic is aimed at creating a multidisciplinary forum of discussion on the recent advances in controlling assistive and rehabilitation robots presenting the diversity of the current approaches. It was expected to include Research Topics related to developments of controllers for upper limb/lower limb robotic exoskeletons, biomechanical investigations of robotic exoskeletons and other assistive robots, novel sensors for exoskeleton robots to generate human-like motions, EMG/EEG signals-based exoskeleton robot control, mathematical and physical algorithms for control of exoskeleton robots, assessment and benchmarking of exoskeleton robots' functionality, and clinical studies of assistive robot control. The Research Topic received overwhelming attention from the relevant researchers. Eleven articles have been selected to be published on this Research Topic after carrying out a rigorous peer-review process. Out of 11 articles, seven articles are based on the use of EMG signals for the control of assistive and rehabilitation robots. They are

- *Toward Hand Pattern Recognition in Assistive and Rehabilitation Robotics Using EMG and Kinematics* of Zhou et al.
- *Multi-Joint Angles Estimation of Forearm Motion Using a Regression Model* of Qin et al.
- *A Multi-Information Fusion Method for Gait Phase Classification in Lower Limb Rehabilitation Exoskeleton* of Zhang et al.
- *Face-Computer Interface (FCI): Intent Recognition Based on Facial Electromyography (fEMG) and Online Human-Computer Interface with Audiovisual Feedback* of Zhu et al.
- *Improved Motion Classification with an Integrated Multimodal Exoskeleton Interface* of Langlois et al.
- *Robust Torque Predictions from Electromyography Across Multiple Levels of Active Exoskeleton Assistance Despite Non-linear Reorganization of Locomotor Output* of George et al.
- *Control of Newly-Designed Wearable Robotic Hand Exoskeleton Based on Surface Electromyographic Signals* of Li et al.

Another three articles are based on the use of EEG signals.

- *Engagement Enhancement Based on Human-in-the-Loop Optimization for Neural Rehabilitation* of Wang et al.
- *Multi-Feature Input Deep Forest for EEG-Based Emotion Recognition* of Fang et al.
- *The Differences Between Motor Attempt and Motor Imagery in Brain-Computer Interface Accuracy and Event-Related Desynchronization of Patients with Hemiplegia* of Chen et al.

The article titled “*Evaluating Convolutional Neural Networks as a Method of EEG–EMG Fusion*” of Tryon and Trejos presented the use of EMG and EEG signals with a hybrid approach to control the assistive and rehabilitation robots.

Out of seven articles discussed on the EMG signals-based control of assistive and rehabilitation robots, in three articles EMG signals have been used for the control of upper-limb rehabilitation robots. Another two of the articles explained the uses of EMG signals for lower-limb exoskeleton robots and the rest of the articles described the classification of EMG signals to use for rehabilitation purposes.

The physiological and neurological differences among individuals can cause divergent responses to the same task, and the responses can further change considerably during training; both of these factors make engagement enhancement a challenge. This challenge can be overcome by training task optimization based on subjects' responses. In one article an engagement enhancement method based on human-in-the-loop optimization is proposed and the performance of the proposed method is demonstrated by the validation and comparison experiments. The results show that both subjects' surface EMG (sEMG) -based motor engagement and electroencephalography based neural engagement can be improved significantly and maintained at a high level.

Another article proposes a feature fusion and decision fusion method that combines EMG features and kinematic features for hand pattern recognition toward application in upper limb assistive and rehabilitation robotics. Ten normal subjects and five post-stroke patients participating in the experiments were tested with eight hand patterns of daily activities while EMG and kinematics were recorded simultaneously.

A prosthetic hand with high accuracy and robustness are necessary to improve the life quality of forearm amputees. The application of sEMG signals to control a prosthetic hand is challenging. The authors of one of the articles have proposed a time-domain Convolutional Neural Network (CNN) model for the regression prediction of joint angles in three degrees of freedom (DoFs) and 5-fold cross-validation was used to evaluate the correlation coefficient. The 3DOFs includes two wrist joint motion and one finger joint motion. In this study, CNN models were developed using combined EEG–EMG inputs to determine if they have potential as a method of EEG–EMG fusion that automatically extracts relevant information from both signals simultaneously. EEG and EMG signals were recorded during elbow flexion-extension and used to develop CNN models based on time-frequency and time-domain image inputs.

It is very important to have a natural, stable, and comfortable human-computer interface for controlling rehabilitation assistance robots to solve problems of patients who have lost limb control ability, such as upper limb amputation and high paraplegia. One article presents a complete limbs-free face-computer interface framework based on facial electromyography including offline analysis and online control of mechanical equipment.

One of the studies covered in this Research Topic explored the accuracy of the Brain-Computer-Interface (BCI) and event-related desynchronization between the two tasks. The articles identified the promise and the challenges faced by the field of assistive and rehabilitation robotic technologies and identified the critical need for additional prospective, in the design and control of relevant robots. Another article demonstrated a new wearable robotic hand exoskeleton with multi joints, higher degrees of freedom, and a larger range of motion. The exoskeleton hand comprises six linear actuators and can realize both independent movements of each digit and coordinative movement involving multiple fingers for grasp and pinch. The kinematic parameters of the hand exoskeleton were analyzed by using a motion capture system.

This Research Topic provides a snapshot of the current status of research on the *EMG/EEG Signals-Based Control of Assistive and Rehabilitation Robots*. All articles underlined both the promise and the challenges faced by the field of assistive and rehabilitation robots. The articles further recognized the need for additional perspective, in the control of these robotic devices. In summary, all the articles presented in the Research Topic have set a new trend in *EMG/EEG Signals-Based Control of Assistive and Rehabilitation Robots*.

## Author Contributions

All authors listed have made a substantial, direct, and intellectual contribution to the work and approved it for publication.

## Conflict of Interest

The authors declare that the research was conducted in the absence of any commercial or financial relationships that could be construed as a potential conflict of interest.

## Publisher's Note

All claims expressed in this article are solely those of the authors and do not necessarily represent those of their affiliated organizations, or those of the publisher, the editors and the reviewers. Any product that may be evaluated in this article, or claim that may be made by its manufacturer, is not guaranteed or endorsed by the publisher.

